# Nature and nurture in fussy eating from toddlerhood to early adolescence: findings from the Gemini twin cohort

**DOI:** 10.1111/jcpp.14053

**Published:** 2024-09-19

**Authors:** Zeynep Nas, Moritz Herle, Alice R. Kininmonth, Andrea D. Smith, Rachel Bryant‐Waugh, Alison Fildes, Clare H. Llewellyn

**Affiliations:** ^1^ Department of Behavioural Science & Health University College London London UK; ^2^ Social, Genetic & Developmental Psychiatry Centre, Institute of Psychiatry, Psychology, and Neuroscience King's College London London UK; ^3^ School of Food Science and Nutrition University of Leeds Leeds UK; ^4^ MRC Epidemiology Unit University of Cambridge Cambridge UK; ^5^ South London and Maudsley NHS Foundation Trust London UK; ^6^ School of Psychology University of Leeds Leeds UK

**Keywords:** Longitudinal studies, twins, eating behaviour

## Abstract

**Background:**

Food fussiness (FF) describes the tendency to eat a small range of foods, due to pickiness and/or reluctance to try new foods. A common behaviour during childhood, and a considerable cause of caregiver concern; its causes are poorly understood. This is the first twin study of genetic and environmental contributions to the developmental trajectory of FF from toddlerhood to early adolescence, and stability and change over time.

**Methods:**

Participants were from Gemini, a population‐based British cohort of *n* = 4,804 twins born in 2007. Parents reported on FF using the Child Eating Behaviour Questionnaire ‘FF’ scale when children were 16 months (*n* = 3,854), 3 (*n* = 2,666), 5 (*n* = 2,098), 7 (*n* = 703), and 13 years old (*n* = 970). A mixed linear model examined the trajectory of FF, and a correlated factors twin model quantified genetic and environmental contributions to variation in and covariation between trajectory parameters. A longitudinal Cholesky twin model examined genetic and environmental influences on FF at each discrete age.

**Results:**

We modelled a single FF trajectory for all children, which was characterised by increases from 16 months to 7 years, followed by a slight decline from 7 to 13 years. All trajectory parameters were under strong genetic influence (>70%) that was largely shared, indicated by high genetic correlations. Discrete age analyses showed that genetic influence on FF increased significantly after toddlerhood (16 months: 60%, 95% CI: 53%–67%; 3 years: 83%; 81%–86%), with continuing genetic influence as indicated by significant genetic overlap across every age. Shared environmental influences were only significant during toddlerhood. Unique environmental influences explained 15%–26% of the variance over time, with some enduring influence from 5 years onwards.

**Conclusions:**

Individual differences in FF were largely explained by genetic factors at all ages. Fussy eating also shows a significant proportion of environmental influence, especially in toddlerhood, and may, therefore, benefit from early interventions throughout childhood. Future work needs to refine the FF trajectory and explore specific trajectory classes.

## Introduction

Food fussiness describes the tendency to eat a limited range of foods, often due to pickiness regarding flavour or texture, and/or the reluctance to try new foods and flavours. FF or picky eating is common and typically develops early in life (during toddlerhood) with prevalence rates ranging between 6% and 50% (Machado, Dias, Lima, Campos, & Gonçalves, 2016; Mascola, Bryson, & Agras, 2010; Taylor, Wernimont, Northstone, & Emmett, 2015). There have been few studies of the developmental trajectory of fussy and picky eating, which suggests the behaviours tend to peak in early‐to‐middle childhood, with most, though not all, children showing decreases in FF as they mature into adolescence (Cardona Cano et al., 2015; de Barse et al., 2015; Herle et al., 2020; Taylor & Emmett, 2019; Taylor, Steer, Hays, & Emmett, 2019). Persistent and/or severe fussiness can contribute to detrimental physical and psychological health outcomes, such as nutritional deficiencies, weight faltering, and anxiety over food (Lafraire, Rioux, Giboreau, & Picard, 2016; Taylor et al., 2015). Severe FF causes considerable caregiver anxiety, can disrupt family mealtimes (Wolstenholme, Heary, & Kelly, 2019), and challenges family dynamics (Zucker et al., 2015). Some epidemiological research has also linked FF in childhood with increased risk for disordered eating in adolescence and young adulthood (Carter Leno, Micali, Bryant‐Waugh, & Herle, 2022; Herle et al., 2020; McClelland, Robinson, Potterton, Mountford, & Schmidt, 2020). Excessive selective eating can be a key symptom of avoidant/restrictive food intake disorder (ARFID), a relatively recently recognised eating disorder included in the DSM‐5 (Zimmerman & Fisher, 2017). In summary, FF is common, potentially important to the healthy development of children, and is often a major cause for concern among caregivers.

Understanding the relative influence of genetic and environmental factors on FF at different ages is key for informing intervention efforts to support caregivers in managing fussy eating behaviours. For example, if environmental influences are stronger at one‐time point, then interventions targeting this age group are likely to have the greatest chance of success. On the other hand, if genetic influences dominate at a particular age, then interventions may be more challenging, targeted, personalised, and more intensive management may be needed, while reassuring parents that they are not to blame. We have previously conducted cross‐sectional twin studies of FF in toddlerhood (16 months) and early childhood (3 years) (Fildes, van Jaarsveld, Cooke, Wardle, & Llewellyn, 2016; Smith et al., 2017), but to date there are no studies of the relative influence of genetic and environmental factors on the developmental course of FF from toddlerhood through to adolescence. In this context, little is known about the aetiology of problematic fussy eating patterns characterised by continued high levels of fussy eating from toddlerhood into adolescence, captured in trajectory analyses, or the changing genetic and environmental influences on FF at different developmental stages.

The main aims of this study are to:

1. Model the developmental trajectory of fussy eating from toddlerhood to early adolescence and estimate genetic and environmental contributions to individual differences in trajectory parameters.
2. Estimate genetic and environmental contributions to fussy eating at different ages from toddlerhood to early adolescence and examine stability and change in these relative contributions to fussy eating from 16 months to 13 years of age.

## Methods

### Sample

Participants were from Gemini, a population‐based cohort of twin children born in England and Wales in 2007 (Jaarsveld, Johnson, Llewellyn, & Wardle, 2010), who have been followed up for over a decade. Gemini participants were recruited through the UK Office for National Statistics, which contacted all eligible families with twins born between March and December 2007 (*n* = 6,754), for consent to participate. Of the 3,435 families who consented, 2,402 completed the baseline questionnaire, and these families constitute the main baseline Gemini cohort. The cohort includes measures capturing growth, eating behaviours, appetite, home environment, and health outcomes. The study was initially granted ethical approval in 2007 through the University College London Committee for the Ethics of non‐National Health Service Human Research, with continuing approval for subsequent data collection waves. In this study, we use data collected from parents when the twins were on average 16 months (*n* = 3,854), 3 years (*n* = 2,666), 5 years (*n* = 2,098), 7 years (*n* = 703) and 13 years of age (*n* = 970).

### Measures

#### Fussy eating

‘FF’ is a subscale of the parent‐reported Child Eating Behaviour Questionnaire (CEBQ) a widely used parent‐reported measure of fussy eating behaviours in children. It has good internal and test–retest reliability (Wardle, Guthrie, Sanderson, & Rapoport, 2001) and has been validated against psychiatric interviews (Steinsbekk, Sveen, Fildes, Llewellyn, & Wichstrøm, 2017), clinical measures of feeding problems (Rogers, Ramsay, & Blissett, 2018) and independent behavioural observation measures of fussy eating in children (Rendall, Dodd, & Harvey, 2020). The scale incorporates both FF (two items) as well as food neophobia (four items). The two constructs are highly correlated (*r* = .72, *p* < .001) and share a common aetiology as previously shown in Gemini at 16 months (Smith et al., 2017). The scale was administered when the children were 16 months, 3, 5, 7, and 13 years old (Wardle et al., 2001). At 7 years, data were only collected in a subsample as part of a targeted round of dietary data collection. The subscale consists of six items and is rated on a five‐point Likert scale from ‘never’ to ‘always’ (example item: ‘My child decides that s/he doesn't like a food, even without tasting it’). At 16 months, an adapted version of the CEBQ (the CEBQ‐T), suitable for toddlers was used, although the items for the FF subscale remain identical. Items included are listed in Table S1.

#### Zygosity, age and sex

Opposite‐sex pairs were classified as dizygotic. The zygosity of same‐sex twin pairs was assigned using a widely used parent‐reported questionnaire measure of similarity (Herle, Fildes, van Jaarsveld, Rijsdijk, & Llewellyn, 2016; Price et al., 2000) that was completed at 8 months and again at 29 months and validated using DNA (for more detail on the process, see Herle et al., 2016). Child sex was parent‐reported at baseline, and age of the twins at each wave was calculated from the parent‐reported date of birth and the date the questionnaires were completed at the included waves.

### Data analyses

#### Longitudinal change in fussy eating – developmental trajectory analysis

We first examined the overall trajectory of fussy eating across the five time points using linear mixed model analysis in Stata. The mixed‐effects framework lends itself to the analyses of repeated measures as it accounts for the nonindependence of measures within an individual. The mixed model estimated three parameters: intercept, linear slope, and quadratic slope. Briefly, the intercept refers to the starting average FF score for the sample, the linear slope describes the pattern of change from the intercept, and the quadratic slope indicates whether this linear pattern changes over time. After model fitting, we extracted parameters using the best linear unbiased predictions (BLUPs). Genetic analyses quantifying the variance and covariance between the three parameters were undertaken using the twin design, as outlined in more detail below.

Analyses were pre‐registered, see here: https://osf.io/jcwsq. We diverged from the pre‐registration in that we originally aimed to derive multiple latent trajectories of fussy eating using a growth mixture model approach. However, models did not converge – potentially due to reduced variability within twin pairs, and hence we were not able to derive meaningful latent trajectories. Instead, we estimated one average trajectory using a mixed model.

#### Genetic analyses

The twin design is based on the known genetic difference between monozygotic (MZ; identical) and dizygotic (DZ; nonidentical) twin pairs; MZ pairs share 100% of their genetic material, whereas DZ pairs share, on average, 50% of their segregating DNA. Using this information, the total phenotypic variance of a trait can be decomposed into three latent components: Additive genetic influences (A); common/shared environmental influences (C); and unique environmental influences (E), which also includes random measurement error. The pattern of twin correlations provides an indication of the genetic and environmental influences on variation in, and covariation between, traits; maximum likelihood structural equation modelling (MLSEM) is used to derive more precise estimates of A, C, and E with 95% confidence intervals and goodness‐of‐fit statistics.

### Twin correlations

Comparisons between correlations for MZ and DZ pairs at each time point provide an indication of the aetiology of the trait at each age. If MZ twin correlations are larger than the DZ twin correlations this indicates a genetic contribution, with larger differences suggesting a larger contribution of genetic differences to the *variance* in that trait. On the other hand, similar‐sized correlations between MZ and DZ twins indicate an important contribution from the shared environment and less genetic influence. The extent to which the MZ correlation is not 1.0 indicates the contribution of unique environmental factors (and random measurement error), which are the only source of differences between MZ twin pairs (because they are matched completely for genetic and shared environmental influences). Within‐twin cross‐time correlations indicate the phenotypic stability in the measured phenotype (e.g. FF) over time (*r*_pheno_). Cross‐twin, cross‐time correlations provide an indication of the likely source of *covariance* across ages (i.e. the extent to which the same genetic or environmental factors contribute to longitudinal stability in FF). In the same way, cross‐twin, cross‐trait correlations provide an indication of the extent to which common genetic or environmental factors contribute to covariation between different traits measured at the same time point (i.e. different trajectory parameters of FF).

### Maximum likelihood structural equation modelling

We fitted a multivariate Correlated Factors Model to examine the genetic and environmental contributions to variation in and covariation between the three parameters of FF (FF) trajectories (intercept, slope, and quadratic terms). This model is appropriate for examining shared aetiology underlying multiple variables that are measured cross‐sectionally, with no particular temporal ordering between them. In this model, the aetiological correlations (additive genetic correlation; rA, common/shared environmental correlation; rC and unique environmental correlation; rE) indicate the extent to which the genetic and environmental influences underlying the different phenotypes are the same. They can be interpreted like a Pearson's correlation: for example, a high positive correlation indicates that many of the same genetic influences that contribute to higher scores on one trait, also influence higher scores on the other trait; a high negative genetic correlation indicates that many of the same genetic influences that contribute to higher scores on one trait, also influence lower scores on the other trait.

We fitted a longitudinal Cholesky Decomposition Model to examine genetic and environmental influences on variation in FF at each of the five ages, and to stability and change in FF over time. A Cholesky Decomposition Model is appropriate for examining the variation in and covariation between longitudinal data – i.e. the same phenotype that has been measured repeatedly at different time points (Rijsdijk & Sham, 2002). A longitudinal ACE Cholesky model is conceptually comparable to a hierarchical regression in that the independent contributions of genetic and environmental influences are assessed after the contributions of previous influences have been accounted for. In the Cholesky decomposition, these are known as unique genetic and environmental influences at each age, independent of influences from previous time points (these effects are estimated by paths a11, a22, a33, c11, c22, …, e11, e22, as demonstrated on the example path diagram shown in Figure S1). Additionally, the method estimates the extent to which genetic and environmental influences carry over from one time point to another (these are known as overlapping aetiological effects, and are estimated by paths a21, a31, a41, etc.). The first aetiological influences (i.e. paths a11, c11, and e11) represent all genetic and environmental influences on the first measured age point (these capture influences that are both unique to this age, as well as those overlapping with previous ages, but for simplicity, we represent these as unique influences in this study).

Genetic analyses were conducted in R using the structural equation modelling package, OpenMX (Neale et al., 2016). The program handles missing data via full‐information maximum‐likelihood estimation (FIML). Sex and ages corresponding to each wave were regressed out from raw data prior to twin modelling analyses, and residuals were analysed. We began by fitting a fully saturated phenotypic model (Gaussian decomposition), which estimated all parameters without constraints. Next, we fitted two submodels, the first constraining means and variances across twin order and zygosity, and the second also constraining the phenotypic correlations to be equated across twin order and zygosity specifying symmetric cross‐twin cross‐trait correlation matrices in MZ and DZ groups. We also extracted the most parsimonious twin model by dropping non‐significant parameters. For instance, if we find that shared environmental effects are non‐significant, we will constrain these non‐significant parameters to zero and test whether this results in a significant reduction in fit.

The goodness‐of‐fit of the models was assessed with minus twice the log‐likelihood (−2LL). Difference in −2LL between a full model and a nested submodel (simpler model with fewer parameters) was assessed by χ^2^ tests and *p‐*values, such that the more parsimonious nested model is preferred only if this does not result in a significant reduction in fit. In addition, we used the Akaike Information Criterion (AIC) and Bayesian Information Criterion as indicators for model fit, with lower values indicating the better fitting model (Posada & Buckley, 2004).

## Results

Descriptive statistics on child (zygosity, age, sex and FF score) and family characteristics (mothers' age at birth, ethnicity, education level at baseline, household income category, and twins' gestational age) at each time wave are provided in Table 1. Nonresponse analyses between the baseline Gemini cohort (8 months) suggest that the 7‐ and 13‐year data are of higher socio‐economic status (SES), feature a larger proportion of White families and mothers are generally older and have a longer gestational period (Table S11). The distribution of FF at the five time points as well as a spaghetti plot can also be viewed in the Supporting Information (Figures S2 and S3). There was no significant reduction in fit from the fully saturated model to the more parsimonious Cholesky and Correlated Factors Models, as indicated by non‐significant changes in χ^2^ and decreasing AIC values (Tables S2 and S3).

**Table 1:** Descriptive statistics

Child variable	Wave				
	16 months	3 years	5 years	7 years	13 years
*n*	3,854	2,666	2,098	703	970
Zygosity	MZ = 1,232 DZ = 2,562 Unknown = 60	MZ = 909 DZ = 1,731 Unknown = 26	MZ = 700 DZ = 1,384 Unknown = 14	MZ = 232 DZ = 471	MZ = 334 DZ = 630 Unknown = 6
Mean child age (*SD*)	15.82 months (1.15)	3.46 (0.27)	5.15 (0.13)	7.18 (0.24)	12.90 (0.71)
Sex (% male)	1,909 (49.5%)	1,321 (49.5%)	1,030 (49.1%)	349 (49.6%)	474 (48.9%)
FF average score (*SD*) (range 1–5)	2.17 (0.70)	2.66 (0.84)	2.77 (0.83)	2.67 (0.81)	2.60 (0.89)
Family variable					
Average annual household income category (1 = Low, 2 = Medium, 3 = High) (%)	1 = 1,114 (29.8) 2 = 1,794 (48.1) 3 = 826 (22.1)	1 = 701 (27.2) 2 = 1,269 (49.1) 3 = 612 (23.7)	1 = 546 (26.9) 2 = 998 (49.1) 3 = 488 (24.0)	1 = 145 (21.1) 2 = 354 (51.5) 3 = 188 (27.4)	1 = 208 (21.9) 2 = 484 (51.1) 3 = 256 (27.0)
Mother's average age at birth in years (*SD*)	33.35 (5.05)	33.62 (4.75)	33.84 (4.76)	34.46 (4.51)	34.25 (4.36)
Gestational age average (weeks) (*SD*)	36.21 (2.47)	36.19 (2.51)	36.26 (2.43)	36.42 (2.32)	36.32 (2.52)
Mother's education level (%)					
Low	740 (19.2)	436 (16.4)	336 (16.0)	59 (8.4)	122 (12.6)
Intermediate	1,378 (35.8)	939 (35.2)	726 (34.6)	206 (29.3)	294 (30.3)
High	1,736 (45.0)	1,291 (48.4)	1,036 (49.4)	438 (62.3)	554 (57.1)
Mothers' ethnicity (%)					
White	3,636 (94.3)	2,534 (95.0)	2,004 (95.5)	669 (95.2)	932 (96.1)
Non‐white	218 (5.7)	132 (5.0)	94 (4.5)	34 (4.8)	38 (3.9)

Income was measured at child aged ~8 months and categorised as follows (high = >£67.5 k high income; medium = £30 k–67.5 k average UK income, low = <£30 k less than average UK income). Maternal education was measured at child age ~8 months and categorised as: low = no qualifications or high school education for example CSE, GCSE, O level; intermediate = vocational qualification or advanced high school education, and high = University‐level education.

### Genetic and environmental influences on food fussiness (FF) trajectory parameters

#### Food fussiness trajectory

**Figure 1:**
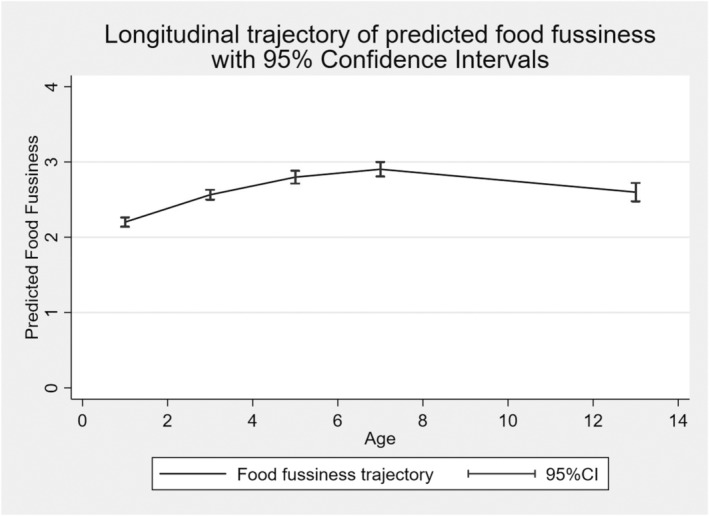
Longitudinal trajectory of mean food fussiness in the Gemini sample from 16 months to 13 years of age

Figure 1 depicts the predicted single trajectory of FF across the five time points, derived from the mixed effects model. Descriptive statistics for the trajectory parameters (intercept, linear and quadratic slopes), along with phenotypic and MZ‐DZ correlations can be found in Supporting Information (Tables S4–S6). The intercept was positively correlated with the linear slope (*r* = .67, 95% CI: 0.65, 0.69), indicating that children who start at a higher average FF score tend to have a larger increase in FF over time. There was a negative correlation between the intercept and quadratic slope, indicating that children who started at a higher FF score also had a steeper decrease of FF over time (*r* = −.82, 95% CI: −0.83, −0.81). There was also a negative correlation between the linear slope and quadratic slope, indicating that children who had a higher linear slope also showed a steeper decrease of FF over time (*r* = −.95, 95% CI = −0.95, −0.94). These findings suggest that those who started at a higher FF score and those with a higher linear increase also showed steeper decreases in fussiness from 7 to 13 years in this trajectory pattern, though fussiness scores remained higher than at starting point on average, highlighting the persistence of FF throughout this period.

##### Twin correlations

Cross‐twin, within trait correlations for MZ pairs for each of the trajectory parameters, were more than twice the size of the DZ pair correlations, suggesting a large genetic contribution to all three parameters. This pattern was similar to the cross‐twin cross‐trait correlations, indicating that the associations between the three trajectory parameters are likely to be genetically driven.

##### Correlated factors model

**Figure 2:**
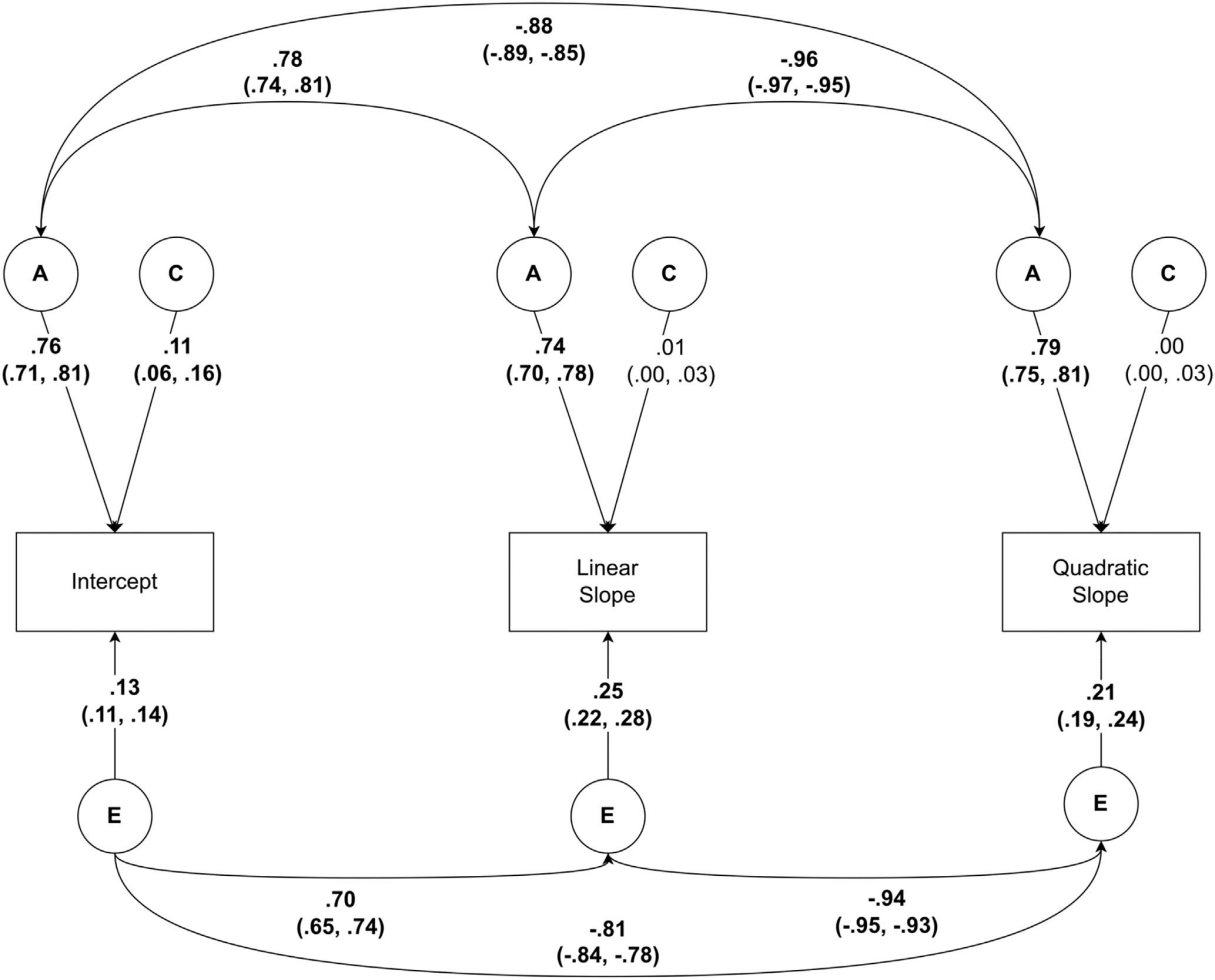
Correlated Factors ACE Model of food fussiness trajectory parameters, including intercept, linear, and quadratic slopes. For simplicity, shared environmental correlations were not reported in this figure, as they were very small and largely non‐significant. Full details of this model can be found in Supporting Information

We found high heritability for all three parameters (Table S7), indicating that the variance in each aspect of the single trajectory of FF is under strong genetic influence. Figure 2 shows the Correlated Factors Model with heritability estimates and aetiological correlations between trajectory parameters (Table S8). We found a large, positive genetic correlation between intercept and linear slope, indicating that many of the same genes that influence a higher starting average FF score also influenced the greater increases from toddlerhood to middle childhood. The genetic correlations between intercept and quadratic slope, and between linear and quadratic slopes were strong and negative, suggesting that many of the same genes that influence higher starting average FF scores, and higher linear increases from toddlerhood to middle childhood also contributed to a steeper decrease in FF from middle childhood to adolescence. In other words, there is a large genetic influence on both the stability and any trajectory changes observed in fussy eating. Unique environmental correlations were of similar magnitude and in the same pattern of direction.

### Genetic and environmental influences on variation in and covariation between food fussiness at each age

#### Twin correlations

Phenotypic correlations between each time point were positive and significant and ranged from moderate to large (*r*_pheno_ = .30–.77) (Table 2), indicating that FF is a moderately to highly stable trait across development. Cross‐twin, within‐time correlations, indicated that genetic influences become more prominent over time because the size of the difference between the MZ and DZ correlations became larger as children matured. Cross‐twin, cross‐time correlations suggested that continuing genetic influences contribute to stability in FF over time, as indicated by larger correlations for MZ than DZ twins (Table S9).

**Table 2:** Pairwise correlation correlations of FF scores (95% CI) measured using the CEBQ

	16 months	3 years	5 years	7 years
3 years	0.45 (0.42, 0.49)			
5 years	0.41 (0.37, 0.45)	0.71 (0.68, 0.73)		
7 years	0.35 (0.29, 0.40)	0.66 (0.62, 0.69)	0.77 (0.74, 0.80)	
13 years	0.30 (0.24, 0.36)	0.52 (0.46, 0.56)	0.63 (0.58, 0.67)	0.70 (0.65, 0.74)

16 months *n* = 3,854; 3 years *n* = 2,666, 5 years *n* = 2,098, 7 years *n* = 703; 13 years *n* = 970.

#### Cholesky decomposition model

We fitted a longitudinal ACE Cholesky Decomposition Model for FF at the five time points. To extract the most parsimonious model, we dropped C paths for ages 3, 5, 7, and 13 years, as these were small and non‐significant. Constraining these paths to zero did not result in a significant reduction in fit (Table S3). However, we retained the C path for FF at 16 months, as this estimate was sizeable and significant (ACE estimates for the full unconstrained model can be viewed in Table S10).

The total contribution of genetic influences on variation in FF (heritability) ranged from 60% to 84% at the five measurement ages, with heritability at 16 months being significantly lower than at all subsequent ages (Table 3). The shared environment was only significant at 16 months and explained 25% of the individual differences in FF at this age. Unique environmental influences explained 15%–26% of the total individual differences in FF across the five measurement points.

**Table 3:** Total variance explained by genetic and environmental influences across time

Time point	A	C	E
16 months	0.60 (0.53, 0.67)	0.25 (0.18, 0.31)	0.15 (0.14, 0.17)
3 years	0.83 (0.81, 0.86)	–	0.17 (0.14, 0.19)
5 years	0.84 (0.80, 0.86)	–	0.16 (0.14, 0.20)
7 years	0.77 (0.70, 0.82)	–	0.23 (0.18, 0.30)
13 years	0.74 (0.67, 0.80)	–	0.26 (0.20, 0.33)

*Note*: A = Additive genetic influences; C = Common environmental influences; E = Unique environmental influences (including measurement error). Results are from constrained ACE model. C influences were dropped due to small, non‐significant estimates at 3,5,7, and 12 years. The total variance in a trait includes both unique influences at that given age as well as influences carried over from previous age(s).

**Figure 3:**
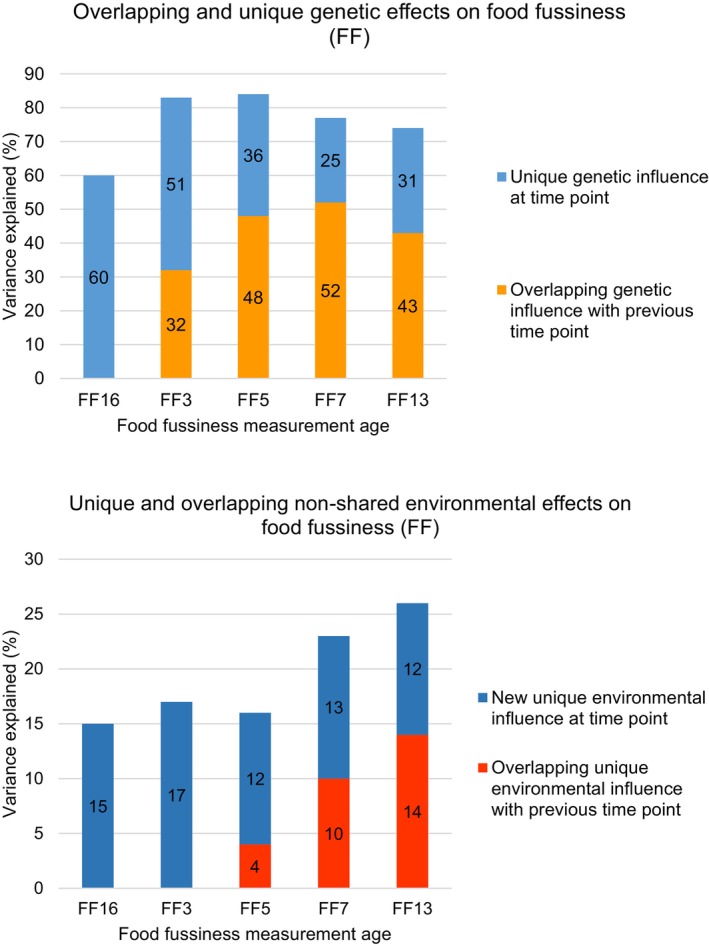
X‐axis represents the five measurements of food fussiness: FF16 = 16 months, FF3 = 3 years, FF5 = 5 years, FF7 = 7 years, FF13 = 13 years. Y‐axis represents the proportion of variance explained by genetic and environmental influences. Shared environmental influences were non‐significant beyond toddlerhood and have, therefore, been omitted from this figure

The Cholesky Decomposition Model also indicated the extent to which the genetic and environmental influences on variation in FF at 3, 5, 7, and 13 years were unique to each age or overlapped with previous ages. Figure 3 summarises the total unique and overlapping A and E influences at each age; Figures S4 and S5 show the path estimates from the fully constrained Cholesky Decomposition Model for A and E influences, respectively. The proportion of genetic influence that was unique to each age decreased over time, while overlapping genetic influences increased cumulatively as children grew older, indicating that common genetic influences contribute strongly to stability in FF over time, in line with the pattern of twin, cross‐twin, cross‐time correlations. Of note, the genetic covariation path between 16 months and 13 years (i.e. the extent to which the genetic influences on FF in adolescence are the same as those already expressed in toddlerhood) was moderate (A Chol = 0.20, 95% CI = 0.13, 0.29) indicating that the genetic influences on FF are both stable and persist over a period of greater than 10 years (Figure S4). However, there was also substantial unique genetic influence at 13 years of age, suggesting new genetic influences on FF come online as the twins reach adolescence. Non‐shared environmental influences also increased over time and influences carried over from previous ages contributed to the stability of FF from 5 to 13 years (overlapping influences were not observed between toddlerhood and 3 years of age) (Figure S5). However, overall, common genetic factors played a far more important role in contributing to stability in FF over time than common unique environmental factors. This was evident from the considerably larger % of total variance in FF at each age explained by overlapping genetic versus overlapping unique environmental factors (3 years: 32% vs. 0%; 5 years: 48% vs. 4%; 7 years: 52% vs. 10%; 13 years: 43% vs. 14%).

## Discussion

This is the first study to establish the genetic and environmental contributions to an overall FF trajectory spanning from toddlerhood to early adolescence and to quantify genetic and environmental influences on individual differences in trait stability and change across five time points.

### Summary of main findings

Modelling of a single longitudinal trajectory revealed FF to be, on average, a highly stable trait. The three trajectory parameters (intercept: i.e. starting average score; linear slope: i.e. rate of linear change; and quadratic slope: i.e. change in the linear growth) were strongly correlated and all under strong genetic influence, with 74%–79% of individual differences explained by genetic factors. Most of the remaining variance in these trajectory parameters was due to non‐shared environmental influences (and measurement error: 13%–25%), with small and mostly non‐significant contributions from the shared environment. We found significant and large genetic and unique environmental correlations between trajectory components of FF. Specifically, there were large and positive genetic and unique environmental correlations between the intercept and linear slope, indicating that the same genetic and unique environmental influences that contributed to a higher average FF score in toddlerhood also contributed to a greater linear increase in FF from toddlerhood to middle childhood. On the other hand, there were large negative aetiological correlations between the linear and quadratic slope, and between the intercept and quadratic slope. This indicated that many of the same genetic and unique environmental influences that contributed to a higher average FF score and greater linear increases in FF from toddlerhood to middle childhood also contributed to the decline in FF from middle childhood to early adolescence. In the context of very high estimates for all three trajectory parameters, these results point towards shared genetic effects being largely responsible for the continuity and persistence of FF throughout childhood and into adolescence, including patterns of persistent and enduring FF.

Discrete age analyses indicated moderate–large phenotypic associations across time, indicating, that FF is a moderately to highly stable trait during child development, in line with the trajectory analyses. We also showed a high heritability of FF at every age from toddlerhood to early adolescence, with the lowest genetic contribution in toddlerhood. At 16 months, genetic factors explained 60% of the variance in FF, which had risen to 74%–84% between the ages of three and 13. Persisting genetic influences that endured throughout development were largely responsible for stability in FF over time. Genetic effects already in operation in toddlerhood continued to have an impact over 10 years later, evidenced by overlapping genetic influences from 16 months to 13 years. In fact, from 5 years of age onwards, about half of the total variance in FF was explained by genetic effects ‘carried over’ from previous time points, with the biggest contribution observed at 7 years. We found that unique environmental influences increased over time, as children transitioned towards greater independence. On the other hand, shared environmental influences were only significant in toddlerhood (25% of variance explained) and were largely negligible by age three.

### Findings in context

Our findings are in line with previous work suggesting that fussy eating and related behaviours can be persistent and enduring for some children (Cardona Cano et al., 2015; Mascola et al., 2010) in line with theory and previous epidemiological studies (Taylor et al., 2015). However, the average trajectory scores never dipped below the initial starting average score, indicating children continued to display more FF at 13 years old than they had at 16 months. Previous work also suggested fussy eating increases and then decreases between 7 and 13 years, though fussy eating scores reached baseline levels at early adolescence, suggesting the need to explore this developmental period further (Carter Leno et al., 2022).

The results from the longitudinal twin modelling align with previous cross‐sectional work in Gemini reporting moderate heritability estimate for FF of 46% at 16 months of age (Smith et al., 2017), and a high heritability estimate of 78% by 3 years of age (Fildes et al., 2016). The current study expands on this earlier work, by embedding a longitudinal approach within a behaviour genetics design. Previous longitudinal work has mainly been phenotypic, such as the association of FF as a risk factor for behaviours like disordered eating, diet quality, and weight status (Antoniou et al., 2016; da Costa et al., 2022; Herle, Stavola, et al., 2020). This study is the first to focus specifically on fussy eating, using repeated measures spanning more than a decade, and offers a unique behaviour genetic context.

The shared environment being significant only in toddlerhood is expected, in line with previous Gemini research (Smith et al., 2017). Another British cohort study indicated that shared environmental influences on variation in food preferences become displaced, and mostly accounted for, by genetics and the unique environment in adulthood (Smith et al., 2016). Research suggests high heritability of ARFID (79%), which features selective eating as a common component, with no significant effect of the shared environment (Dinkler et al., 2023). The high genetic component of FF aligns with that observed for other appetitive traits such as food responsiveness and satiety responsiveness in infancy and late childhood (Carnell, Haworth, Plomin, & Wardle, 2008; Llewellyn, van Jaarsveld, Johnson, Carnell, & Wardle, 2010). For broader context, this finding is also in the range reported by studies of disordered eating in adolescents suggesting moderate–high heritability estimates varying between 28% and 83% (Thornton, Mazzeo, & Bulik, 2011). Genetic differences in the population, therefore, appear to play a particularly important role in determining individual differences in many different eating behaviour phenotypes in infancy, toddlerhood, and adolescence.

### Implications

These findings are important for our understanding of fussy eating in children for three main reasons. First, children's fussy eating is a major cause of concern for caregivers, who often blame themselves, or are blamed by others, for their child's restricted diets or food rejection. This study indicates that FF is under strong genetic influence, which can remain influential throughout childhood. This finding may help to alleviate parental blame and explain why siblings raised in the same environment often express very different selective eating behaviours. Second, the shared environment was only found to significantly impact individual differences in fussy eating behaviour in toddlerhood, indicating environmental or family‐based interventions targeting children's fussy eating behaviours (such as repeated exposure and increasing the variety of fruits and vegetables offered in the home), may be most effective in the very early years (Kamarudin et al., 2023). These findings do not imply that FF cannot be changed in response to behavioural interventions; however, they suggest that it may be a more challenging behaviour to modify in comparison to behaviours that are under predominantly environmental influence. It is also worth noting that the unique environment is found to be stable and steadily increasing over time, implicating that interventions for FF could be implemented across childhood and into adolescence. Third, FF is a stable trait as indicated by moderate‐to‐high phenotypic correlations over time, and sizeable continuing genetic influences driving stability across development. Fussy eating behaviours are not necessarily just a ‘phase’, but potentially follow a persistent trajectory. These findings suggest that toddlers who present with higher FF are also more likely to experience greater increases in FF as they mature. This is of interest not only to researchers but also to clinicians and the wider child and adolescent health community. Given the links between fussy eating and other physical and psychological health outcomes, including eating disorders such as ARFID (Dovey, 2018), early detection and intervention for FF in toddlerhood may reduce the expression of this behaviour across development.

### Future directions

This is the most comprehensive longitudinal twin study of fussy eating in childhood to date. We tested a single trajectory for FF in this paper, and future work may provide more refined insight into the potential trajectory classes over time. There are ongoing efforts to understand the genetic architecture of fussy eating, including genome‐wide analyses (Abdulkadir et al., 2022). However, such studies require large sample sizes and fussy eating – and eating behaviours in general – are not commonly measured in big cohort studies with genomic data, making these approaches challenging. Another important line of enquiry is the phenotypic and genetic associations between fussy eating and other neurodevelopmental disorders in childhood. Previous research has indicated that food neophobia (fear of new foods, closely related to fussy eating) was elevated in children with higher autistic‐like traits (Wallace, Llewellyn, Fildes, & Ronald, 2018) and that fussy eating might be a mediator on the commonly observed path between autism spectrum disorder traits in childhood and risk for disordered eating in adolescence (Carter Leno et al., 2022; Solmi et al., 2021). The relationship between fussy eating and other neurodevelopmental conditions, such as ADHD and mood and anxiety disorders, has also been documented and is worth exploring further (Thorsteinsdottir, Olafsdottir, Brynjolfsdottir, Bjarnason, & Njardvik, 2022; Wu et al., 2023). Last, these findings provide new insights into the complexity of FF and the need for compassionate, evidence‐based interventions and guidance to support caregivers feeding children with varying levels of FF.

### Strengths and limitations

As with most longitudinal studies, attrition over time can contribute to lower statistical power. In this study, there were fewer participants at age seven than at other ages as only a subsample of highly engaged participants were invited to take part in this wave of data collection; this could have resulted in a change in the variance–covariance structure in the multivariate Cholesky model. Although the study spans a large age range, FF can also shift throughout adolescence and into adulthood, prompting replication at other age points. Although the FF items remained identical throughout the different time points, we acknowledge that the scale measures fussy eating at different developmental points and, therefore, may capture a slightly different underlying construct at each stage. We recommend that measurement invariance in the scale is examined further in future studies. Twin studies also rely on key modelling assumptions, such as the ‘equal environments assumption’, which assumes that MZ and DZ twins share environments to a similar extent, and although debated, the assumption has generally been supported (Felson, 2014). FF was parent‐reported and parents' knowledge of their twins' zygosity might influence the ratings of their twins' behaviours, for example parents may be inclined to rate their twins more similarly because they believe them to be identical. However, we examined this in Gemini using the misclassified twin design and parental zygosity perception did not influence their reporting of fussy eating (Herle et al., 2016). The Gemini sample is fairly homogenous, including a large proportion of White‐British households of higher socio‐economic positions compared to the general population. Most characteristics at each wave remain similar descriptively, though, upon further inspection, data at ages 7 and 13 years are less representative of the baseline Gemini cohort. We, therefore, suggest taking findings with this into consideration and recommend replication in more diverse populations. Considering this, to meaningfully advance the field, future research requires large‐scale, genetically sensitive, and longitudinal analyses of fussy eating. Replication is particularly warranted in non‐western populations where food culture, parental feeding practices and food security may vary considerably.

## Conclusions

This novel longitudinal examination provides evidence of FF being a highly heritable trait that is relatively stable from toddlerhood into early adolescence, with genetic influences largely responsible for its continuity.

**Key points**

- What's known: FF is a heritable eating behaviour trait that is common in early childhood and can limit dietary diversity. FF can be challenging for parents to manage and has important implications for health and development, as well as psychosocial implications such as family well‐being and psychological distress.
- What's new: FF is a stable trait that peaks in middle childhood and declines slightly thereafter. It endures into early adolescence, with individual variation in FF remaining under moderate–large genetic influence across development, from toddlerhood into adolescence.
- What's relevant: Parents are not to blame for their children's innate fussy eating behaviours. Interventions targeting FF could start as early as toddlerhood and may need to be tailored and intensive at different developmental time points.

## Supporting information


**Figure S1.** Path diagram for full Multivariate Cholesky twin model.
**Figure S2.** Distributions of food fussiness.
**Figure S3.** Spaghetti plot of food fussiness across waves.
**Figure S4.** Path diagram depicting genetic paths from constrained longitudinal Cholesky model.
**Figure S5.** Path diagram depicting unique environmental paths from constrained longitudinal Cholesky model.
**Table S1.** Food fussiness items.
**Table S2.** Correlated factors twin model fit statistics (intercept, linear slope and quadratic slope).
**Table S3.** Longitudinal Cholesky model fit statistics (discrete age analysis).
**Table S4.** Descriptive statistics for intercept, linear slope and quadratic slope for food fussiness.
**Table S5.** MZ ‐ DZ correlations obtained from correlated factors twin model for intercept, linear slope, and quadratic slope.
**Table S6.** Phenotypic correlations between intercept and linear‐quadratic slopes.
**Table S7.** ACE components for intercept, linear and quadratic slopes.
**Table S8.** Aetiological correlations between intercept and linear‐quadratic slopes.
**Table S9.** Longitudinal Cholesky model MZ and DZ correlations obtained from constrained phenotypic model (Sub model 1b).
**Table S10.** Standardised ACE estimates at each wave obtained from full longitudinal Cholesky model.
**Table S11.** Nonresponse analyses between baseline Gemini cohort and 7‐ and 13‐year data.

## Data Availability

The data that support the findings of this study are available from the corresponding author upon reasonable request.
