# Supplementary material for: Nature and nurture in fussy eating from toddlerhood to early adolescence: findings from the Gemini twin cohort
Source: J Child Psychol Psychiatry. 2024 Sep 19;66(2):241–52. doi: 10.1111/jcpp.14053 (PMC11754699; doi:10.1111/jcpp.14053)
Supplement: Supplementary file 1 — Figure S1. Path diagram for full Multivariate Cholesky twin model. Figure S2. Distributions of food fussiness. Figure S3. Spaghetti plot of food fussiness across waves. Figure S4. Path diagram depicting genetic paths from constrained longitudinal Cholesky model. Figure S5. Path diagram depicting unique environmental paths from constrained longitudinal Cholesky model. Table S1. Food fussiness items. Table S2. Correlated factors twin model fit statistics (intercept, linear slope and quadratic slope). Table S3. Longitudinal Cholesky model fit statistics (discrete age analysis). Table S4. Descriptive statistics for intercept, linear slope and quadratic slope for food fussiness. Table S5. MZ ‐ DZ correlations obtained from correlated factors twin model for intercept, linear slope, and quadratic slope. Table S6. Phenotypic correlations between intercept and linear‐quadratic slopes. Table S7. ACE components for intercept, linear and quadratic slopes. Table S8. Aetiological correlations between intercept and linear‐quadratic slopes. Table S9. Longitudinal Cholesky model MZ and DZ correlations obtained from constrained phenotypic model (Sub model 1b). Table S10. Standardised ACE estimates at each wave obtained from full longitudinal Cholesky model. Table S11. Nonresponse analyses between baseline Gemini cohort and 7‐ and 13‐year data. [file JCPP-66-241-s001.docx]

**Nature and nurture in fussy eating throughout development from toddlerhood to early adolescence: findings from the Gemini twin cohort**

**Supporting information**

Figure S1. Path diagram for full Multivariate Cholesky twin model


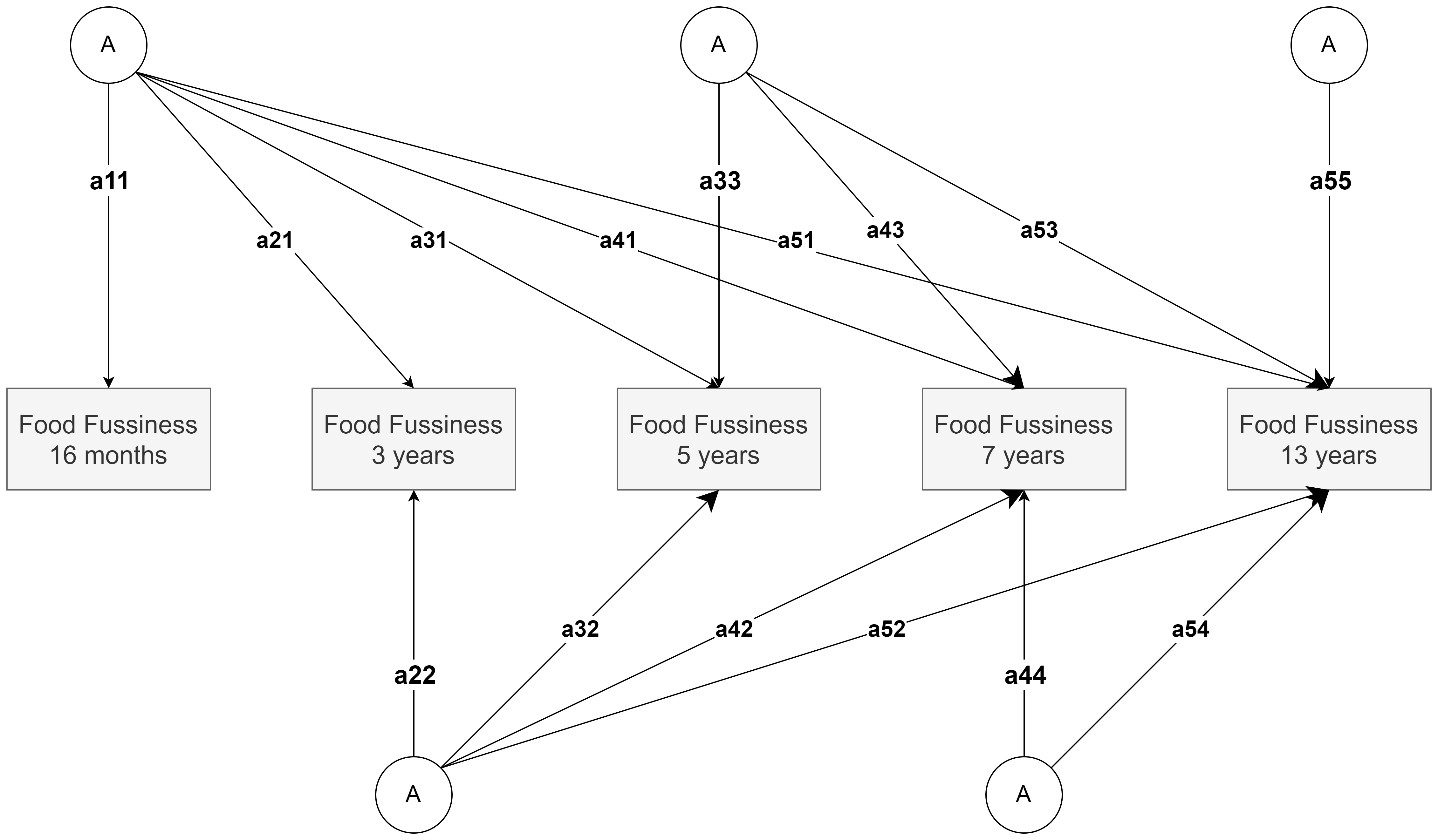


A= Additive genetic influences. Rectangular boxes represent the measured phenotype (food fussiness). Circles indicate latent influences on the measured phenotype. Paths a11, a22, a33, a44 and a55 represent unique genetic influences independent of influences from previous timepoints. Paths a21, a31, a41… etc represent overlapping genetic influences from previous time points. Diagram represents genetic paths only. The same principle applies for illustrating unique and overlapping environmental influences.

Table S1. Food fussiness items

| 1 | My child refuses new foods at first |
| --- | --- |
| 2 | My child enjoys tasting new foods (R) |
| 3 | My child enjoys a wide variety of foods (R) |
| 4 | My child is difficult to please with meals |
| 5 | My child is interested in tasting food he/she hasn’t tasted before (R) |
| 6 | My child decides that s/he doesn’t like a food, even without tasting it |

(R) = reverse coded

Figure S2. Distributions of food fussiness

Figure S3. Spaghetti plot of food fussiness across waves

*
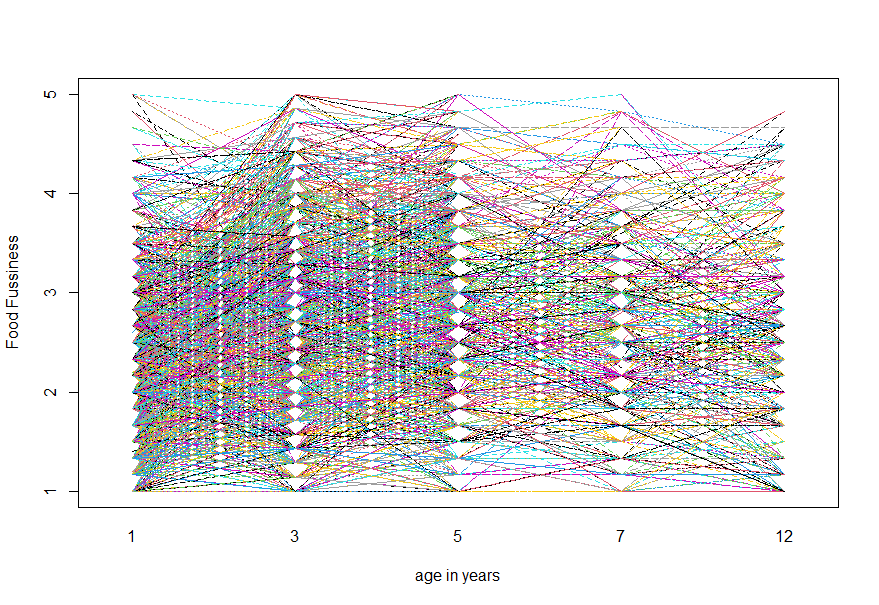
*

Table S2. Correlated factors twin model fit statistics (intercept, linear slope and quadratic slope)

| Model no. | Model | Compared to model | ep | df | -2LL | AIC | BIC | Δ χ² (df) | p-value |
| --- | --- | --- | --- | --- | --- | --- | --- | --- | --- |
| 1 | Full Saturated | - | 54 | 11352 | -9546.18 | -9438.18 | -9138.47 | - | - |
| 2 | Sub model 1a | 1 | 42 | 11364 | -9531.16 | -9447.16 | -9214.05 | 15.02 (12) | 0.24 |
| 3 | Sub model 1b | 1 | 21 | 11385 | -9495.60 | -9453.60 | -9337.04 | 50.58 (33) | 0.03 |
| 4 | Full ACE | - | 21 | 11385 | -9446.24 | -9404.24 | -9287.69 | - | - |

Sub model 1a = means and variances equated across twin order; Sub model 1b = means, variances and phenotypic correlations equated across twin order and zygosity + symmetric cross-twin cross trait correlations within MZ and DZ groups. ep = estimated parameters; df = degrees of freedom; -2LL = Minus two log likelihood; AIC = Akaike’s Information Criterion; BIC = Bayesian Information Criterion; Δ χ² = Difference in chi-squared value. Note that although sub model 1b had a significant reduction in fit, the AIC was the lowest and was therefore preferred. Sub 1a and Sub1b models compared to the fully saturated model.

Table S3. Longitudinal Cholesky model fit statistics (discrete age analysis)

|  | Model | Compared to | ep | df | -2LL | AIC | BIC | Δ χ² (df) | p-value |
| --- | --- | --- | --- | --- | --- | --- | --- | --- | --- |
| 1 | Full Saturated | - | 130 | 9809 | 49780.49 | 50040.49 | 50790.40 | - | - |
| 2 | Sub model 1a | 1 | 110 | 9829 | 49797.53 | 50017.53 | 50652.07 | 17.04 (20) | 0.65 |
| 3 | Sub model 1b | 1 | 50 | 9889 | 49869.03 | 49969.03 | 50257.45 | 88.53 (80) | 0.24 |
| 4 | Full Cholesky ACE | - | 50 | 9889 | 49902.53 | 50002.53 | 50290.96 | - | - |
| 5 | Constrained ACE | 4 | 36 | 9903 | 49919.13 | 49991.13 | 50198.79 | 16.59 (14) | .28 |

Sub model 1a = means and variances equated across twin order; Sub model 1b = means, variances and phenotypic correlations equated across twin order and zygosity + symmetric cross-twin cross trait correlations within MZ and DZ groups. Constrained ACE = Dropping C paths for 3,5,7 and 12 years. ep = estimated parameters; df = degrees of freedom; -2LL = Minus two log likelihood; AIC = Akaike’s Information Criterion; BIC = Bayesian Information Criterion; Δ χ² = Difference in chi-squared value.

Table S4. Descriptive statistics for intercept, linear slope and quadratic slope for food fussiness

| **Variable** | **Mean** | **SE** | **S.D.** | **Min** | **Max** | **95% CI** |
| --- | --- | --- | --- | --- | --- | --- |
| **Intercept** | 2.67 | 0.02 | 0.55 | 1.25 | 4.64 | 2.63, 2.70 |
| **Linear slope** | 0.13 | 0.00 | 0.07 | -0.11 | 0.43 | 0.12, 0.13 |
| **Quadratic slope** | -0.02 | 0.00 | 0.01 | -0.05 | 0.01 | -0.02, -0.01 |

Table S5. MZ - DZ correlations obtained from correlated factors twin model for intercept, linear slope, and quadratic slope.

| **Correlations** | **Variables** | **MZ (95% CI)** | **DZ (95% CI)** |
| --- | --- | --- | --- |
| **Cross twin,**  **within trait** | Intercept | 0.88 (0.86, 0.89) | 0.43 (0.38, 0.47) |
|  | Linear slope | 0.77 (0.74, 0.79) | 0.27 (0.21, 0.32) |
|  | Quadratic slope | 0.80 (0.77, 0.82) | 0.30 (0.25, 0.35) |
| **Cross twin,**  **cross trait** | Intercept- Linear slope | 0.56 (0.53, 0.59) | 0.18 (0.13, 0.22) |
|  | Intercept – Quadratic slope | -0.69 (-0.71, -0.67) | -0.27 (-0.32, -0.23) |
|  | Linear-Quadratic slope | -0.74 (-0.77, -0.72) | -0.26 (-0.31, -0.21) |

Table S6. Phenotypic correlations between intercept and linear-quadratic slopes.

|  | **Intercept** | **Linear slope** |
| --- | --- | --- |
| **Linear slope** | 0.67 (0.65, 0.69) |  |
| **Quadratic slope** | -0.82 (-0.83, -0.81) | -0.95 (-0.95, -0.94) |

Table S7. ACE components for intercept, linear and quadratic slopes

| **Variable** | **A** | **C** | **E** |
| --- | --- | --- | --- |
| **Intercept** | 0.76 (0.71, 0.81) | 0.11 (0.06, 0.16) | 0.13 (0.11, 0.14) |
| **Linear slope** | 0.74 (0.70, 0.78) | 0.01 (0.00 0.03) | 0.25 (0.22, 0.28) |
| **Quadratic slope** | 0.79 (0.75, 0.81) | 0.00 (0.00, 0.03) | 0.21 (0.19, 0.24) |

A=Additive genetic influences, C= common/shared environmental influences, E= Unique/non-shared environmental influences.

Table S8. Aetiological correlations between intercept and linear-quadratic slopes

| Variables | **rA** | **rC** | **rE** | **rPh** |
| --- | --- | --- | --- | --- |
| Intercept- Linear slope | **0.78**  **(0.74, 0.81)** | **-0.99**  **(-0.99, -.96)** | **0.70**  **(0.65, 0.74)** | **0.68**  **(0.66, 0.70)** |
| Intercept – Quadratic slope | **-0.88**  **(-0.89, -0.85)** | -0.99  (-1,1) | **-0.81**  **(-0.84, -0.78)** | **-0.82**  **(-0.83, 0.81)** |
| Linear-Quadratic slope | **-0.96**  **(-0.97, -0.95)** | 0.99 (-1, 1) | **-0.94**  **(-0.95, -0.93)** | **-0.95**  **(-0.95, -0.94)** |

rA= Genetic correlation, rC= Shared environmental correlation, rE= Unique environmental correlation, rPh= phenotypic correlation obtained from the ACE model.

Table S9. Longitudinal Cholesky model MZ and DZ correlations obtained from constrained phenotypic model (Sub model 1b)

| **Correlation** | **Time point(s)** | **MZ (95% CI)** | **DZ (95% CI)** |
| --- | --- | --- | --- |
| **Cross twin, within time** | 16 months | 0.85 (0.83, 0.87) | 0.58 (0.54, 0.61) |
|  | 3 years | 0.84 (0.84, 0.86) | 0.39 (0.33, 0.44) |
|  | 5 years | 0.85 (0.82, 0.87) | 0.33 (0.26, 0.39) |
|  | 7 years | 0.79 (0.73, 0.84) | 0.27 (0.20, 0.34) |
|  | 13 years | 0.78 (0.71, 0.83) | 0.23 (0.13, 0.32) |
| **Cross twin, cross time** | 16 months - 3 years | 0.45 (0.42, 0.49) | 0.27 (0.22, 0.31) |
|  | 16 months - 5 years | 0.39 (0.35, 0.43) | 0.25 (0.20, 0.29) |
|  | 16 months- 7 years | 0.35 (0.28, 0.41) | 0.21 (0.14, 0.27) |
|  | 16 months - 13 years | 0.34 (0.28, 0.41) | 0.16 (0.10, 0.23) |
|  | 3 years - 5 years | 0.64 (0.60, 0.67) | 0.27 (0.21, 0.32) |
|  | 3 years - 7 years | 0.58 (0.53, 0.63) | 0.24 (0.17, 0.31) |
|  | 3 years - 13 years | 0.45 (0.39, 0.51) | 0.15 (0.08, 0.22) |
|  | 5 years - 7 years | 0.65 (0.60, 0.69) | 0.27 (0.20, 0.34) |
|  | 5 years - 13 years | 0.57 (0.51, 0.62) | 0.21 (0.14, 0.28) |
|  | 7 years - 13 years | 0.57 (0.50, 0.62) | 0.16 (0.08, 0.24) |

Table S10. Standardised ACE estimates at each wave obtained from full longitudinal Cholesky model

| **Time point** | **A** | **C** | **E** |
| --- | --- | --- | --- |
| 16 months | **0.53 (0.46, 0.61)** | **0.32 (0.24, 0.38)** | **0.15 (0.13, 0.17)** |
| 3 years | **0.81 (0.76, 0.84)** | 0.03 (0.00, 0.08) | **0.16 (0.14, 0.19)** |
| 5 years | **0.80 (0.75, 0.84)** | 0.04 (0.00,0.09) | **0.16 (0.14, 0.19)** |
| 7 years | **0.75 (0.66, 0.81)** | 0.03 (0.00, 0.10) | **0.22 (0.17, 0.29)** |
| 13 years | **0.74 (0.67, 0.80)** | 0.00 (0.00, 0.03) | **0.26 (0.20, 0.33)** |

**Figure S4.** Path diagram depicting genetic paths from constrained longitudinal Cholesky model.


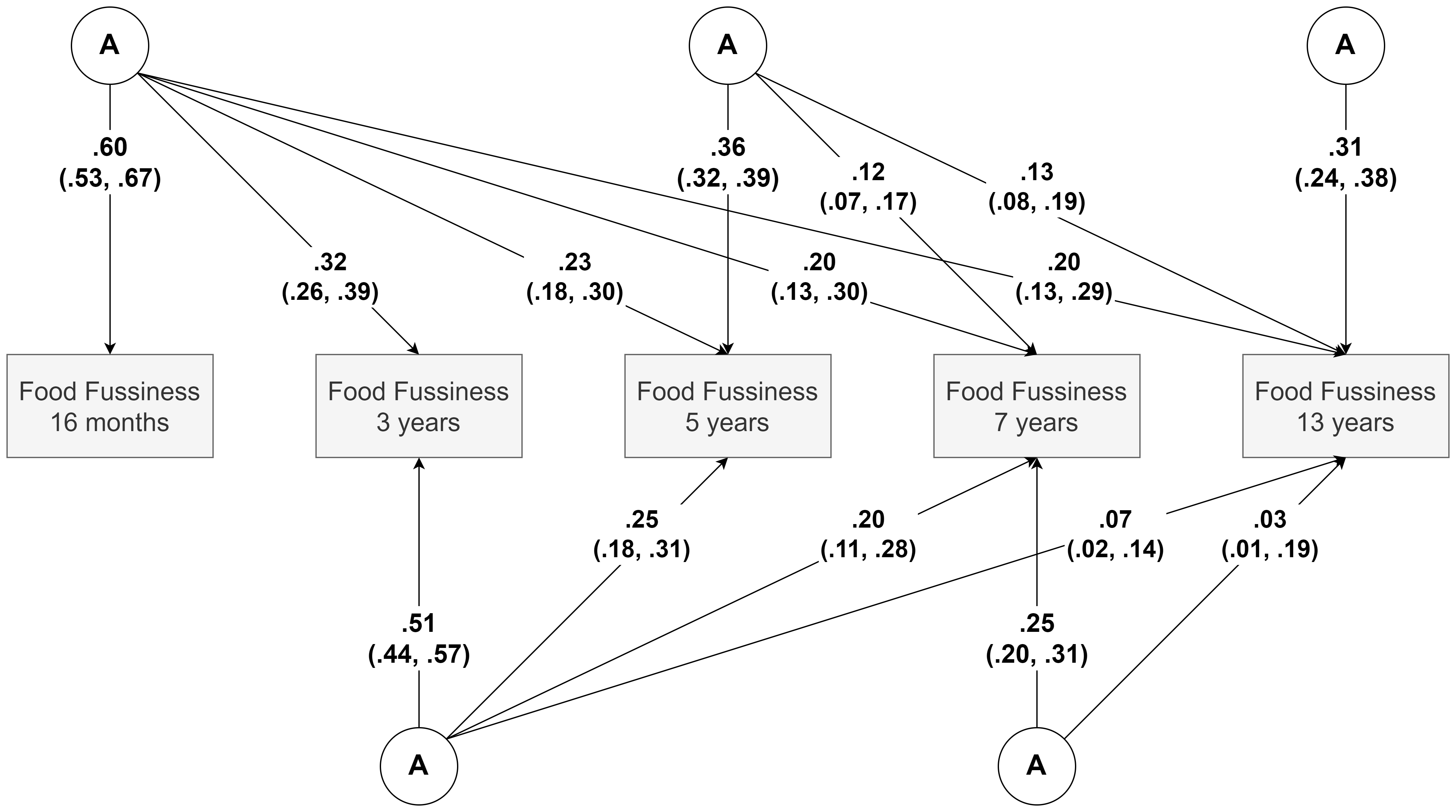


A= Additive genetic influences. Rectangular boxes represent the measured phenotype (food fussiness). Circles indicate latent influences on the measured phenotype. The total variance in food fussiness at each age explained by additive genetic factors can be obtained by summing all paths connecting to that trait at that given age (e.g., total variance in food fussiness explained by additive genetic influences at age 3 is obtained by totalling .32 + .51= .83 (83%)). Total variance components are detailed in Table 3 in the manuscript.

**Figure S5.** Path diagram depicting unique environmental paths from constrained longitudinal Cholesky model.


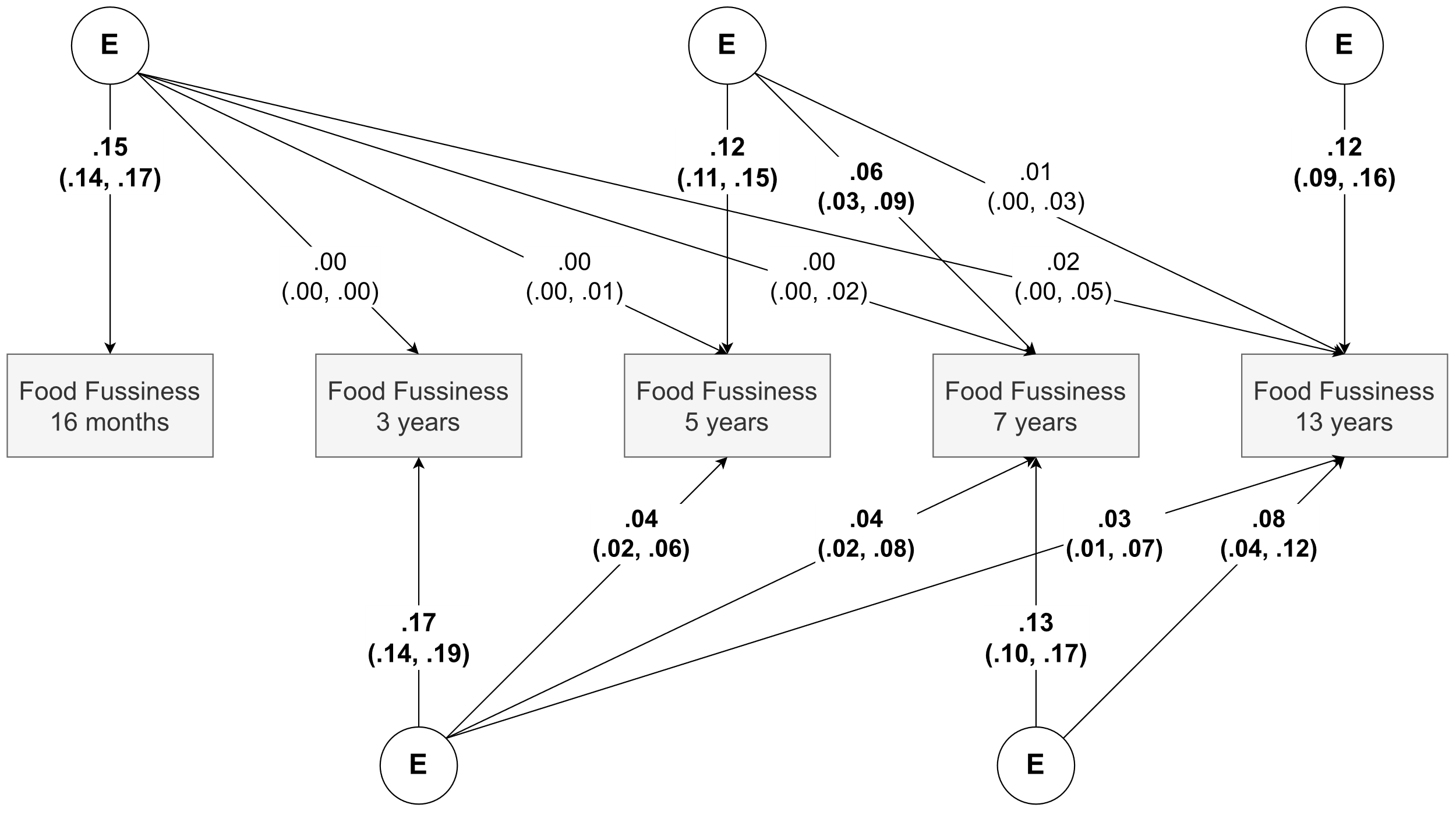


E= Unique environmental influences. Rectangular boxes represent the measured phenotype (food fussiness). Circles indicate latent influences on the measured phenotype. The total variance in food fussiness at each age explained by unique environmental factors can be obtained by summing all paths connecting to that trait at that given age (e.g., total variance in food fussiness explained by unique environmental influences at age 3 is obtained by totalling .00 + .17= .17 (17%)). Total variance components are detailed in Table 3 in the manuscript.

Table S11. Non-response analyses between baseline Gemini cohort and 7- and 13- year data.

| Variable | Baseline-7-year comparisons | | | | | Baseline-13-year comparisons | | | | |
| --- | --- | --- | --- | --- | --- | --- | --- | --- | --- | --- |
|  | 8 months (baseline)  N | N (%) or mean (SD) | 7 years  N | N (%) or mean (SD) | X^2^ / t-test (df), p-value | 8 months (baseline) N | N (%) or mean SD | 13 years  N | N (%) or mean (SD) | X^2^ / t-test (df), p-value |
| Household income | | | | | | | | | | |
| Low | 3941 | 1413 (35.9) | 687 | 145 (21.1) | 62.30 (2), <.001 | 3680 | 1350 (36.7) | 948 | 1=208 (21.9) | 80.72 (2), <.001 |
| Medium |  | 1770 (44.9) |  | 354 (51.5) |  |  | 1640  (44.6) |  | 2=484 (51.1) |  |
| High |  | 758 (19.2) |  | 188 (27.4) |  |  | 690  (18.7) |  | 3=256 (27.0) |  |
| Maternal education | | | | | | | | | | |
| Low | 4101 | 977 (23.8) | 703 | 59 (8.4) | 160.11(2), <.001 | 3834 | 914  (23.9) | 970 | 122 (12.6) | 125.82 (2), <.001 |
| Intermediate |  | 1550 (37.8) |  | 206  (29.3) |  |  | 1462 (38.1) |  | 294 (30.3) |  |
| High |  | 1574 (38.4) |  | 438  (62.3) |  |  | 1458  (38.0) |  | 554 (57.1) |  |
| Maternal ethnicity | | | | | | | | | | |
| White | 4097 | 3793 (92.6) | 703 | 669 (95.2) | 5.73(1), .02 | 3830 | 3530 (92.2) | 970 | 932 (96.1) | 17.53 (1), <.01 |
| Non-white |  | 304 (7.4) |  | 34 (4.8) |  |  | 300  (7.8) |  | 38 (3.9) |  |
| Gestational age | 4081 | 36.2 (2.51) | 703 | 36.42 (2.32) | -2.64 (1005.2), <.01 | 3814 | 36.17 (2.47) | 970 | 36.32 (2.52) | -1.66 (1480), p=.10 |
| Maternal age at birth | 4089 | 32.69 (5.25) | 703 | 34.46 (4.51) | -9.36 (1057.8),  <.001 | 3822 | 32.62 (5.33) | 970 | 34.25 (4.36) | -9.90 (1777.1), p<.001 |

Baseline is child age ~8 months. Income was measured at child aged ~8 months and categorized as follows (high = >£67.5k high income; medium = £30k > average UK income, low= <£30k less than average UK income). Maternal education was measured at child age ~8months and categorised as: low = no qualifications or high school education e.g., CSE, GCSE, O level; intermediate= vocational qualification or advanced high school education, and high = University-level education.

P-values reflect differences by characteristic between the final study sample and the non-response sample and were obtained by chi-square tests or t-tests.
